# Delta-CT radiomics based model for predicting postoperative anastomotic leakage following radical resection of esophageal squamous cell carcinoma

**DOI:** 10.3389/fonc.2024.1485323

**Published:** 2024-10-14

**Authors:** Huantian Li, Linjun Zhang, Lina Song, Yong Wang, Ping Song, Yingjian Ye, Xiumei Li, Peng An

**Affiliations:** ^1^ Department of Surgery and Radiology, Xiangyang No.1 People’s Hospital, Hubei University of Medicine, Xiangyang, China; ^2^ Department of Oncology, Pathology and Epidemiology, Xiangyang Key Laboratory of Maternal-fetal Medicine on Fetal Congenital Heart Disease, Xiangyang No. 1 People’s Hospital, Hubei University of Medicine, Xiangyang, Hubei, China

**Keywords:** post thoracotomy pulmonary infection (PTPI), radiomics, nomogram, least absolute shrinkage and selection operator (LASSO), esophageal squamous cell carcinoma (ESCC), anastomotic leakage (AL)

## Abstract

**Objective:**

To predict postoperative anastomotic leakage (AL) following radical resection of esophageal squamous cell carcinoma (ESCC) based on clinical data and preoperative enhanced Computed tomography(CT) radiomics of the esophagus.

**Method:**

We retrospectively analyzed the clinicopathological and radiological data of 213 patients with ESCC who received radical resection at Xiangyang No.1 People’s Hospital from July 2011 to February 2024. 3D slicer software was used in combination with Lasso extraction and 10-fold cross-validation to extract texture parameters from contrast-enhanced CT images and generate Delta-Radscores. Several models were built using logistic regression to predict postoperative AL in ESCC.

**Results:**

In the training set, the univariate analysis confirmed that duration of surgery, surgical method, delta radscore 1, delta radscore 2, contrast enhancement patterns, peripheral lymph node metastasis, post thoracotomy pulmonary infection(PTPI), and hot pot were risk factors for ESCC-AL (P<0.05 for both). The multivariate analysis showed that delta radscore 1, delta radscore 2, PTPI, and hot pot were independent risk factors for AL (P<0.05 for all). These results were verified by the XGboost machine learning model. The combinational model based on all of the above risk factors [AUC 0.900, OR 0.0282, 95%CI 0.841-0.943] outperformed either the clinical model[AUC 0.759, OR 0.0392, 95%0.683-0.825,P<0.05] or the imaging model[AUC 0.869, OR 0.0335, 95%0.804-0.918,P=0.1277] alone in predictive efficacy. The decision curve proved that the combinational model had a higher clinical net benefit. The nomogram generated via the combinational model simplified the predictive process. The same predictions were verified in the testing set.

**Conclusion:**

Delta radscore 1, delta radscore 2, PTPI, and hot pot were related to ESCC-AL. The novel nomogram created using enhanced CT radiomics informed perioperative management and improved the survival quality of ESCC patients.

## Introduction

**Figure 1:**
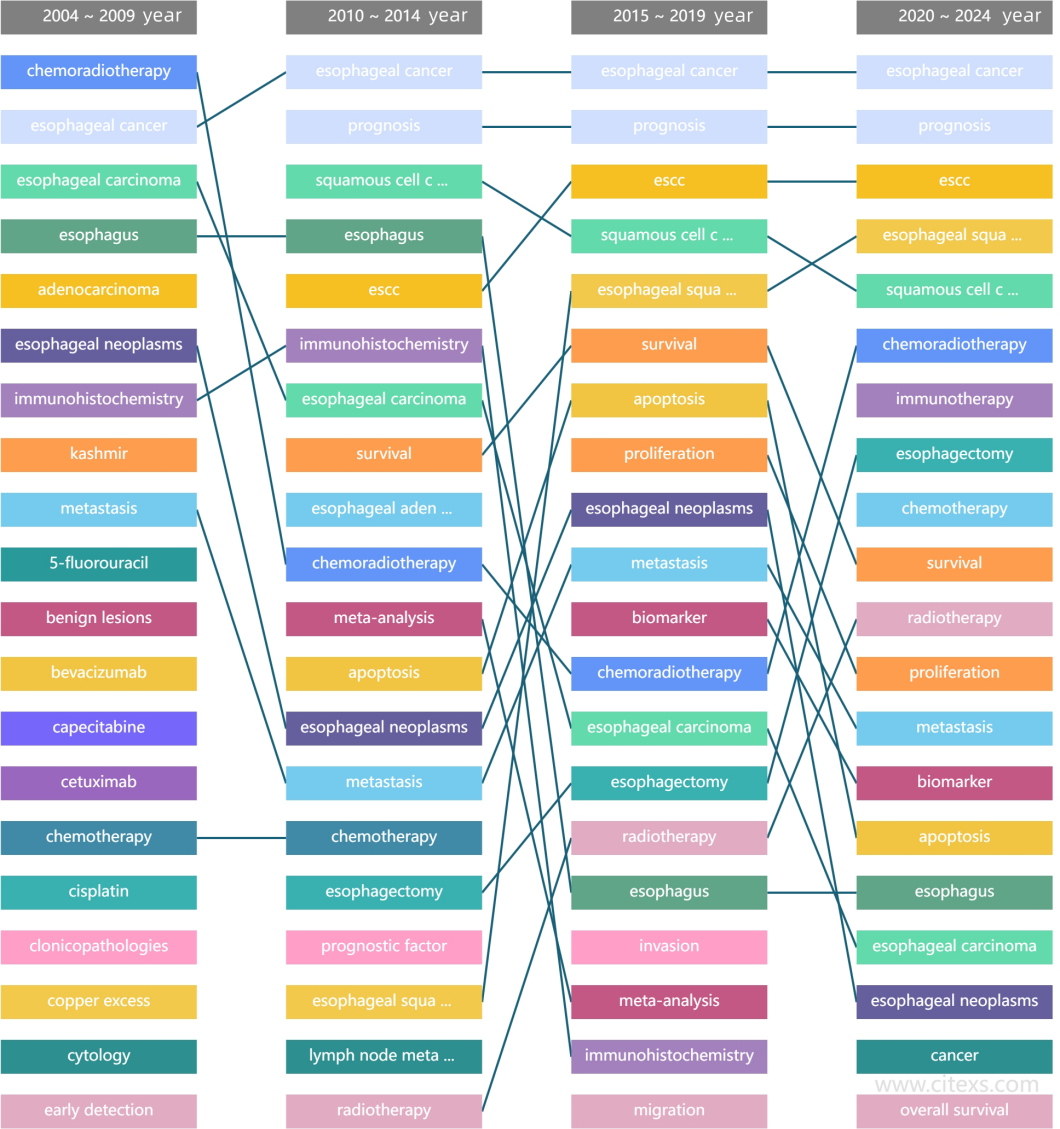
Referring to Pubmed, in recent years, the hot topics of esophageal squamous cell carcinoma had focused on molecular mechanism, chemoradiotherapy and surgical methods. There were rare reports on the ESCC-AL prediction using clinical radiomics data.

China has witnessed a deterioration of environmental and food pollutions in recent years, accompanied by a significant rise in the incidence of esophageal carcinoma (90% of the cases are esophageal squamous cell carcinomas (ESCC)), which reaches 15.87/100,000. The incidence of esophageal carcinoma is even higher, reaching 30/100,000 and far above the global average, in Henan Province and Sichuan Province, where Spicy Hot Pot and pickled foods are more popular than in other parts of China. ESCC is an aggressive malignant carcinoma arising from the esophageal squamous epithelial cells and easily infiltrates into the mediastinum and causes lymph node metastases. ESCC poses a serious threat to human health and has poor prognosis and high mortality (1–3). At present, treatments for ESCC include endoscopy, neoadjuvant chemotherapy plus surgical treatment, and palliative radiotherapy. Along with the progress of surgical techniques, the modified Ivor-Lewis surgery, Sweet surgery, laparothoracoscopy plus minimally invasive esophagectomy (MIE) have been widely applied in clinical practice. These treatments have the benefits of shorter surgical duration, smaller intraoperative blood loss, fewer complications, and shorter ICU stay compared with traditional open thoracotomy. However, the incidence of postoperative AL remains high, and the perioperative management for AL is still challenging. Therefore, developing a novel predictive tool for ESCC-AL is an urgent task (4, 5). Radiomics is an emerging image post-processing technology that extracts texture features of tumors from medical images in a high-throughput manner and describes intratumoral heterogeneity and biological behaviors of tumors. Radiomics makes up for the defects of conventional diagnostic imaging and provides valuable data for developing individualized treatment decisions for a variety of tumors. It has been reported that radiomics can correctly predict the degree of peritumoral invasion and lymphovascular invasion (LVI), which is critical for making surgical options, accelerating postoperative rehabilitation, and reducing AL (6–8). We checked the content of PubMed website about ESCC, and most of the studies focused on the molecular mechanism, chemoradiotherapy and surgical methods of ESCC, etc. there was no report on the prediction of ESCC-AL by radiomics (**Figure 1**). This study focused on predicting the risk of postoperative ESCC-AL based on preoperative delta enhanced CT radiomics to optimize surgical strategies and improve the survival rate and survival quality of ESCC patients after surgery.

## Materials and methods

### Subjects

**Figure 2:**
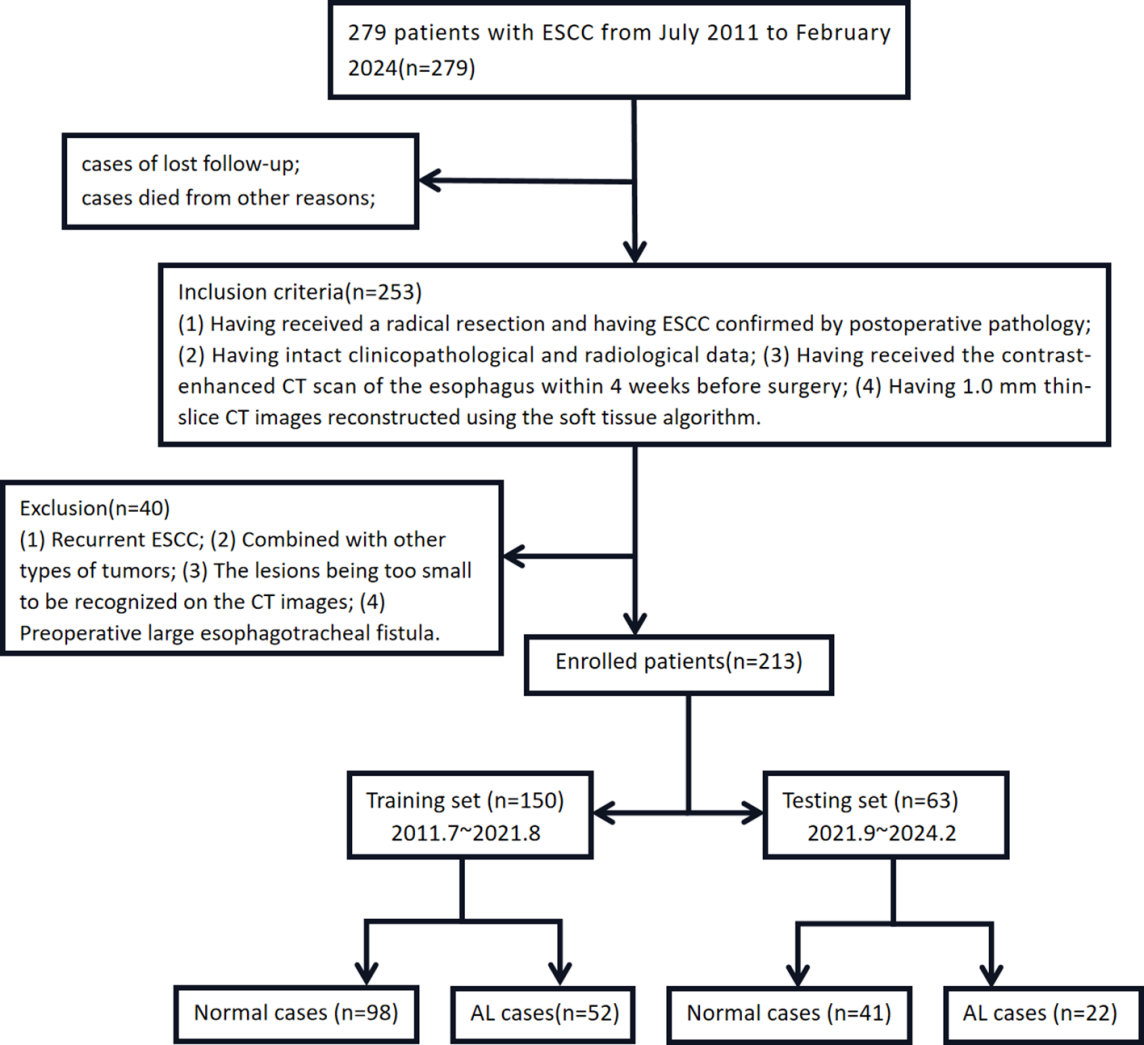
The case registration and grouping sketch map of this study. Notes: the training set cases were sourced at the East District (Headquarters) of Xiangyang NO.1 People’s Hospital from July 2011 to August 2021 and were modeled in the original version. The testing set cases were sourced at the West District (Dongjin Hospital) of Xiangyang NO.1 People’s Hospital from September 2021 to February 2024 and were externally validated to reduce bias.

213 ESCC patients who received radical resection at Xiangyang No.1 People’s Hospital from July 2011 to February 2024 were recruited. The demographic, clinicopathological, and radiological features were extracted from the patients’ medical records. Clinical staging was performed according to the 7^th^ edition of the AJCC TNM staging system for esophageal cancer. There were 114 males (53.52%) and 99 females (46.47%), who were aged 55.75 ± 10.71 years old. Inclusion criteria: (1) Having received a radical resection and having ESCC confirmed by postoperative pathology; (2) Having intact clinicopathological and radiological data; (3) Having received the contrast-enhanced CT scan of the esophagus within 4 weeks before surgery; (4) Having 1.0 mm thin-slice CT images reconstructed using the soft tissue algorithm. Exclusion criteria: (1) Recurrent ESCC; (2) Combined with other types of tumors; (3) The lesions being too small to be recognized on the CT images; (4) Preoperative large esophagotracheal fistula. The ESCC patients were divided into the training set and the test set in a 7:3 ratio and the time cut-off point (**Figure 2**). The present study was approved by the ethics committee of the Xiangyang No.1 People’s Hospital, and all enrolled patients gave their written informed consent (9, 10).

This exploratory, single-center, retrospective study was conducted at our hospital after obtaining approval from the Institutional Review Board (Approval No. XYYYE20240011). The study followed the principles outlined in the Declaration of Helsinki. Written informed consent was obtained from all participants or their guardians. The study also adhered to the STROBE guidelines for reporting. The patient details were deidentified to protect their confidentiality (8–10).

### Surgical treatments

Depending on the size and position of ESCC and the results of negotiation with the patients, the patients received the modified Ivor-Lewis surgery, Sweet surgery, or Combined thoracoscopic and laparoscopic minimally invasive esophagectomy (MIE), as appropriate. The tubes were indwelled for gastrointestinal decompression after surgery, followed by enteral nutrition therapy for 3-5 days using a nasogastric tube or a jejunostomy tube. The intrathoracic drain tubes were removed 5-7 days after surgery. The patients were allowed to drink a small amount of water for 5-7 days after surgery in the absence of discomfort and main complaints and ate a liquid and semi-liquid diet afterwards. The patients took conventional acid-suppressing drugs after surgery, and gastric motility drugs were given, if necessary. All patients were first followed up at 4 weeks after discharge and then once every 3 months (11–13).

### Diagnosis of AL

The patients had persistent fever, cough and vomiting. The position and size of AL were finally confirmed by chest CT, gastrografin esophagography, use of methylene blue reagent, or gastroscopy. The possibility of thoracogastric fistula or perforation at the non-anastomotic site was excluded when making the diagnosis (14).

### Examination method

All ESCC cases were examined using the Siemens dual-source CT scanner(Somatom Definition Flash,Germany) after fasting for 6 h before the scan. They were told to drink 500-1000 ml of purified water within 3 min before the scan to ensure the cleanness and dilatation of the esophageal lumen. The patients took a supine position, head first into the gantry. Images were acquired using the end-expiration breath-holding technique. The scanned area extended from the oropharyngeal opening to the greater curvature of the stomach. The scan parameters were as follows: Tube voltage 120 kV, automatic tube current modulation technique, slice thickness 1.0 mm, rotation time 0.5 s, 512×512 matrix, pitch 1.2, B40f reconstruction algorithm, and reconstruction thickness 1.0 mm. Iohexol (concentration 300 mgI/mL, injection rate 3.0-4.0 ml/s, dose 1.5 mL/kg) was injected via the elbow vein using the high-pressure injection pump. Arterial and venous phase scans were performed with a 30 s delay, followed by the injection of 50 ml of normal saline at the same flow rate (15).

### Contrast-enhanced CT image segmentation and radiomics feature extraction

**Figure 3:**
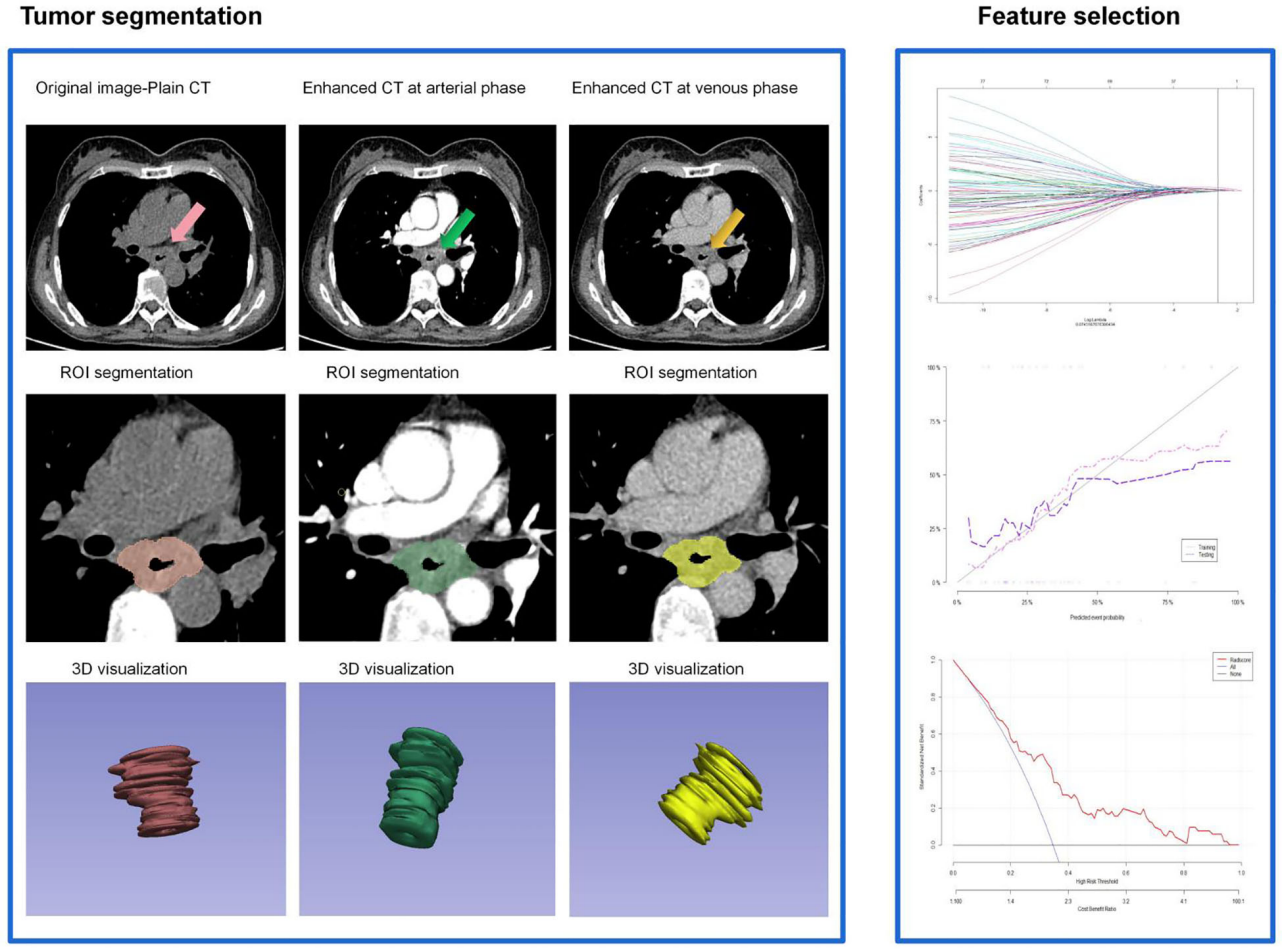
Flow-process diagram of the delta enhanced CT radiomics extracted and radscore generated in this study.

Tumor region of interest (ROI) was outlined on the arterial phase 1.0 mm thin-slice CT images using 3D Slicer (version 4.11) through multi-layer segmentation. Tumors were recognized according to the following criterion: Abnormal thickening of the esophageal wall by above 5 mm, or the esophageal lumen diameter> 10 mm (closed), with local irregular narrowing. Manual ROI selection encompassed the intratumoral necrotic area, but not the gases and liquid within the esophageal lumen, peripheral adipose tissues, heart and lung tissues, or bone tissues. The manually delineated tumor ROI was then revised by reference to the multi-plane reconstruction images. Tumor ROI was segmented manually by two thoracic radiologists with 10 years of experience. Intra-class correlation coefficient (ICC) was used to assess the consistency between the segmentation parameters obtained by two radiologists and the repeatability of the extracted radiomic features. An ICC above 0.75 indicated high consistency and reproducibility of the extracted radiomics features, and ICC≥0.75 was required in our study. Radiological features were extracted using a plug-in(informatics-radiomics) within the 3D slicer. Before that, the images were first subjected to resampling and grayscale discretization to standardize the CT images. A total of 1065x3 radiomics features were extracted, including first-order features, shape features, texture features, and higher-order features. The following steps were adopted to identify the optimal radiomic features: First, radiomics features showing statistically significant differences between the patients with and without AL were identified using the Wilcoxon rank-sum test (P<0.1). Next, those with an intra-unit Correlation Coefficient above 0.9 were removed using the Spearman’s correlation analysis. Finally, LASSO and 10-fold cross-validation were performed, and Radscores were generated based on the remaining features (**Figure 3**). Delta Radscore 1=Radscore in the arterial phase-Radscore in the plain scan phase, Delta Radscore2=Radscore in the arterial phase-Radscore in the venous phase, Delta Radscore3=Radscore in the venous phase-plain scan phase (16, 17).

### Statistical method

All statistical analyses were performed using SPSS 24.0 and R 4.1.3. The measurements that obeyed a normal distribution were presented as mean ± standard deviation (X ± s) and analyzed by the t-test. Those not obeying a normal distribution were analyzed by the rank-sum test. Enumeration data were compared using the chi-square test or Fisher’s exact test. Risk factors for ESCC-AL were analyzed by logistic regression in the training set, and several predictive models were built. The predictive efficacy of the risk factors was tested using the XGboost machine learning model. The AUC values of the models were compared using the Delong test, and the models’ net benefits were compared using the decision curve. All findings were verified in the test set. Novel nomogram and calibration curves were created and used in clinics. P<0.05 was taken to indicate a significant difference (18).

## Results

1. Seventy-four patients (74/213, 34.7%) experienced AL after the surgeries. The univariate analysis in the training set confirmed that the gender distribution, ASA classification, prior thoracic surgery, chronic respiratory disease history, hypertension history, diabetes history, smoking history, drinking history, age, FEV1%, BMI, tumor location, TNM, degree of tumor differentiation, PLR, NLR did not differ significantly between the two groups (P>0.05). The duration of surgery, surgical method, delta radscore 1, delta radscore 2, contrast enhancement patterns, peripheral lymph node metastasis, PTPI, hot pot were significantly different between the two groups (P<0.05). The multivariate analysis suggested that the delta radscore 1, delta radscore 2, PTPI, hot pot were independent risks factor for ESCC-AL. The above results were verified using the XGboost machine learning model. The SHAP value indicated correlations between delta radscore 1, delta radscore 2, PTPI, hot pot and ESCC-AL (**Tables 1**–**4**) (P<0.05 for all).

**Table 1:** Comparison results of clinical and imaging data between two groups.

Factors	AL group	Normal group	*X^2^* or *t* value	P
Gender			1.280	0.200
Male	24	56		
Female	28	42		
ASA classification			1.507	0.134
I	15	34		
II	7	24		
III	30	40		
Prior thoracic surgery			0.208	0.836
1(No)	39	75		
2(have previous thoracic surgery)	13	23		
Chronic respiratory disease history			1.035	0.302
1(No)	37	78		
2(Occasional or mild symptoms)	10	13		
3(Frequent or severe symptoms)	5	7		
Hypertension history	13.518 ± 14.801	16.668 ± 14.886	1.236	0.218
Diabetes history	10.555 ± 6.372	9.647 ± 4.880	0.872	0.384
Smoking history	21.608 ± 15.633	20.228 ± 15.440	0.519	0.605
Drinking history	25.673 ± 17.748	24.577 ± 20.013	0.332	0.741
Age	57.915 ± 10.828	56.205 ± 8.145	1.088	0.278
FEV1%	68.550 ± 7.982	68.791 ± 8.351	0.171	0.865
BMI	26.072 ± 4.395	26.889 ± 3.811	1.185	0.238
Tumor location			1.192	0.235
1(Lower esophagus)	22	52		
2(Upper esophagus)	12	19		
3(Middle lower esophagus)	18	27		
TNM			0.912	0.363
0-1 stage	23	52		
2 stage	11	17		
3 stage	18	29		
Duration of surgery	254.077 ± 32.593	242.755 ± 25.715	2.334	0.021*
Surgical method			2.032	0.044*
1(Ivor-Lewis surgery)	8	5		
2(Sweet surgery)	12	21		
3(MIE)	32	72		
Radscore 1	2.676 ± 0.702	2.082 ± 0.497	6.007	<0.05*
Radscore 2	3.520 ± 0.980	2.594 ± 0.626	7.041	<0.05*
Contrast enhancement patterns			2.209	0.029*
1(Outflow type)	31	72		
2(Plateau type)	13	21		
3(Inflow type)	8	5		
Peripheral lymph node metastasis			2.160	0.032*
0(No)	24	63		
1(Yes)	28	35		
PTPI			3.814	<0.05*
1(No)	26	75		
2(Mild symptoms)	15	18		
3(Severe symptoms)	11	5		
Hot pot			4.674	<0.05*
0(Hardly)	14	64		
1(Occasionally,≤5times/month)	33	31		
2(Often,>5times/month,most even>10)	5	3		
Degree of tumor differentiation			0.747	0.456
1(Well differentiated)	28	59		
2(Poorly differentiated)	24	39		
PLR	16.479 ± 6.717	15.377 ± 4.903	1.148	0.253
NLR	14.423 ± 6.073	12.835 ± 4.530	1.810	0.072

ASA classification, American Society of Anesthesiologists classification; MIE, laparothoracoscopy plus minimally invasive esophagectomy; PTPI, post thoracotomy pulmonary infection; NLR, neutrophil-to-lymphocyte ratio; PLR, platelet lymphocyte ratio. * represents P<0.05.

**Table 2:** Logistic regression analysis results of clinical model based on clinical characteristics for predicting the ESCC-AL, *P < 0.05.

Clinical Model	Univariate analysis		Multivariate analysis	
factors	P	Hazard ratio	P	Hazard ratio
Gender	0.135	1.359(0.909-2.032)		
ASA classification	0.134	1.349(0.912-1.997)		
Prior thoracic surgery	0.835	1.087(0.497-2.380)		
Chronic respiratory disease history	0.302	1.324(0.777-2.256)		
Hypertension history	0.217	0.986(0.963-1.011)		
Diabetes history	0.383	1.015(0.982-1.049)		
Smoking history	0.602	1.006(0.984-1.028)		
Drinking history	0.739	1.003(0.985-1.021)		
Age	0.277	1.021(0.983-1.061)		
FEV1%	0.864	0.996(0.956-1.041)		
BMI	0.237	0.950(0.873-1.031)		
Tumor location	0.234	1.264(0.860-1.858)		
TNM	0.361	1.194(0.817-1.745)		
Duration of surgery	0.024*	1.014(1.001-1.027)		
Surgical method	0.047*	1.681(1.006-2.809)		
PTPI	<0.05*	2.486(1.491-4.145)	0.002*	2.517(1.423-4.454)
Hot pot	<0.05*	3.780(2.012-7.103)	<0.05*	3.571(1.816-7.023)
Degree of tumor differentiation	0.453	1.297(0.658-2.557)		
PLR	0.253	1.036(0.975-1.105)		
NLR	0.077	1.062(0.993-1.134)		

**Table 3:** Logistic regression analysis results of imaging model based on imaging characteristics for predicting the ESCC-AL, *P < 0.05.

Imaging Model	Univariate analysis		Multivariate analysis	
factors	P	Hazard ratio	P	Hazard ratio
Delta Radscore 1	<0.05*	5.722(2.771-11.818)	<0.05*	4.871(2.257-10.512)
Delta Radscore 2	<0.05*	4.098(2.451-6.852)	<0.05*	4.250(2.379-7.593)
Contrast enhancement patterns	0.032*	1.756(1.050-2.937)		
Peripheral lymph node metastasis	0.034*	2.101(1.060-4.162)		

**Table 4:** Logistic regression analysis results of combinational model based on amentioned characteristics for predicting the ESCC-AL, *P < 0.05.

Combined Model	Univariate analysis		Multivariate analysis	
factors	P	Hazard ratio	P	Hazard ratio
Delta Radscore 1	<0.05*	5.722(2.771-11.818)	<0.05*	5.635(2.236-14.202)
Delta Radscore 2	<0.05*	4.098(2.451-6.852)	<0.05*	5.062(2.526-10.145)
Contrast enhancement patterns	0.032*	1.756(1.050-2.937)		
Peripheral lymph node metastasis	0.034*	2.101(1.060-4.162)		
Duration of surgery	0.024*	1.014(1.001-1.027)		
Surgical method	0.047*	1.681(1.006-2.809)		
PTPI	<0.05*	2.486(1.491-4.145)	0.03*	2.459(1.093-5.531)
Hot pot	<0.05*	3.780(2.012-7.103)	<0.05*	9.135(2.643-31.572)

**Figure 4:**
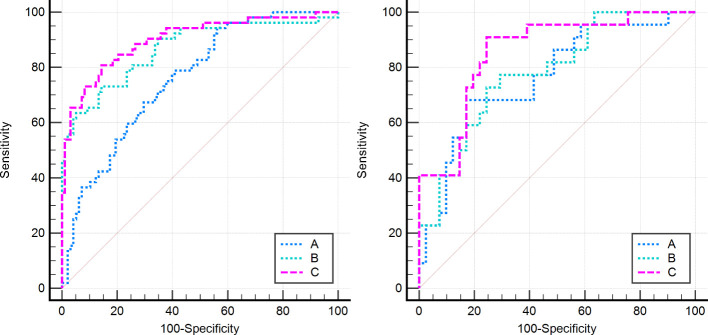
All models in this research were compared by the Delong curve. The left figure showed the training set and the right figure showed the testing set. (A) The clinical model, (B) the imaging model, and (C) the combinational model.

**Figure 5:**
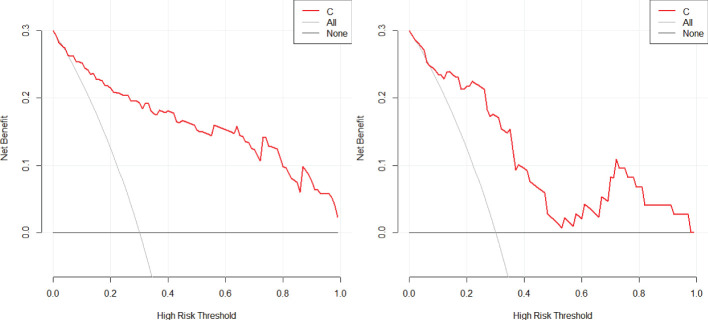
The decision curve analysis of the combined model with the training (left) and testing sets (right) in this study confirmed that the combinational model had a higher clinical net benefit.

**Figure 6:**
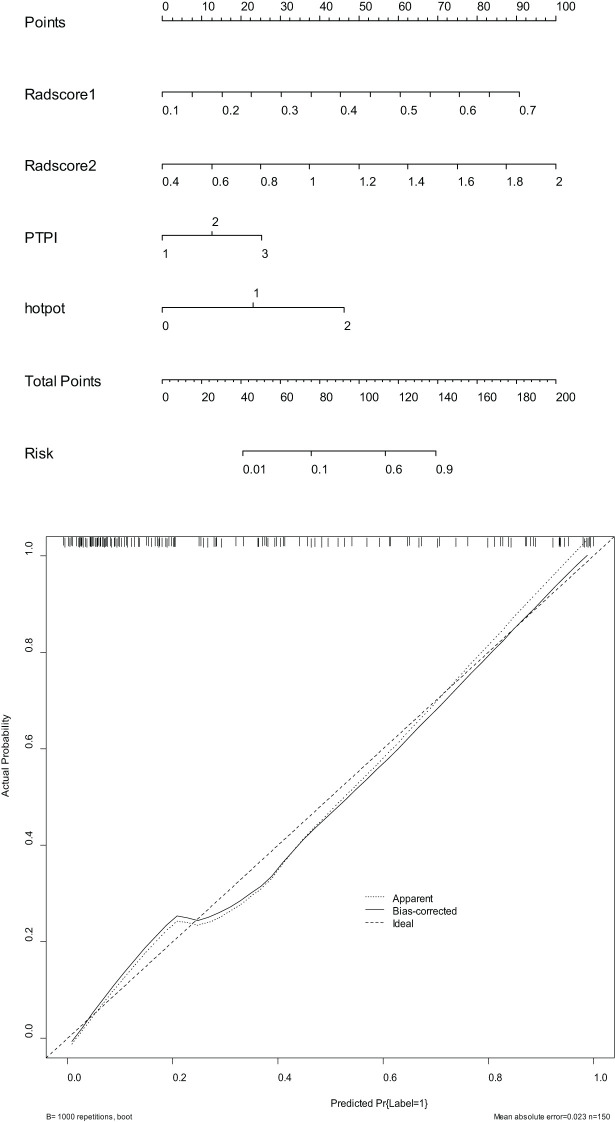
The nomograms (above) and calibration curves (below) developed based on the combinational model have been well evaluated in clinical trials.

**Figure 7:**
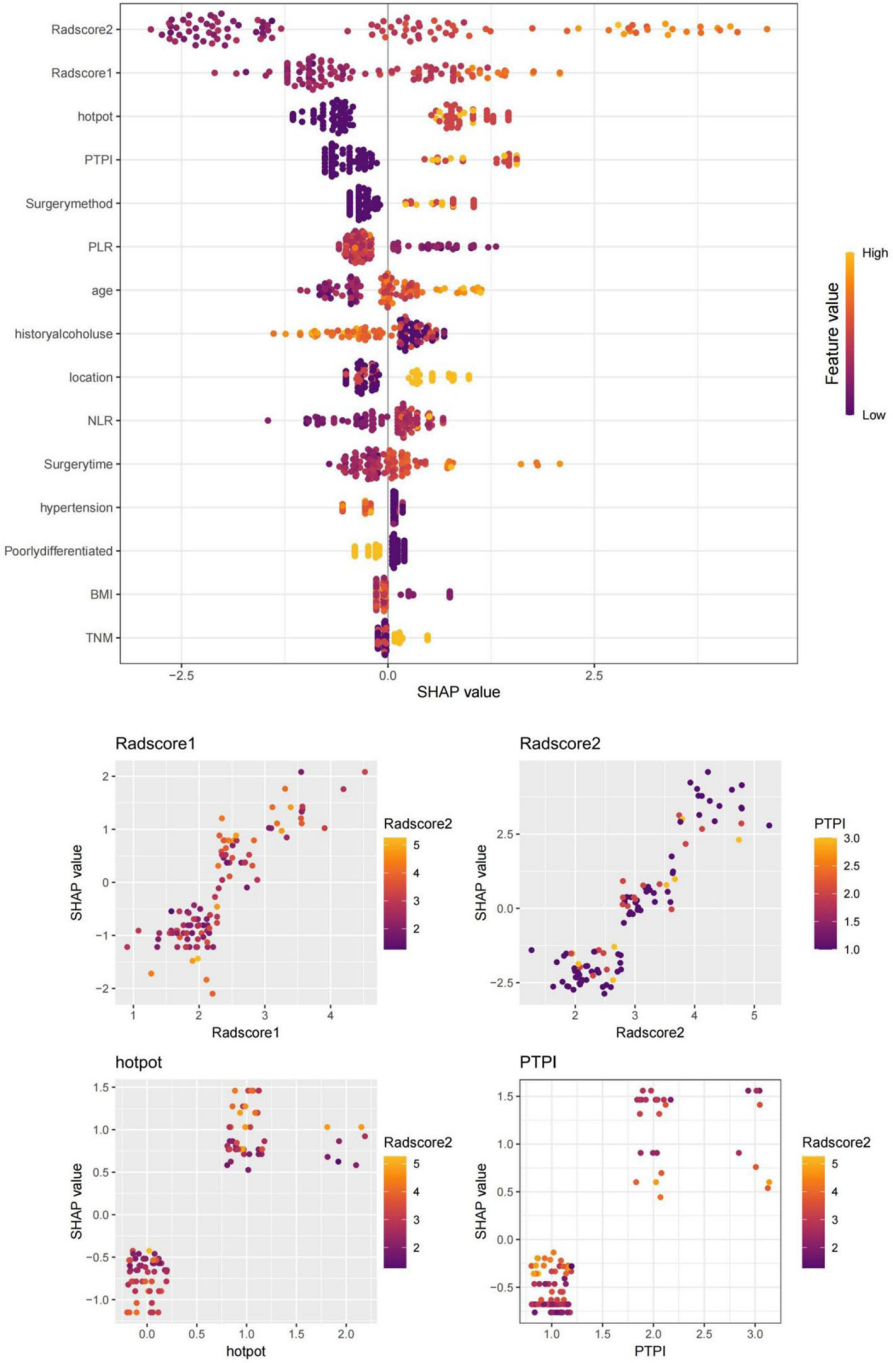
SHAP values of the XGBoost algorithm model output. SHAP values confirmed that delta radscore 1, delta radscore 2, PTPI, hot pot, and so forth, were important influencing factors for predicting ESCC-AL, consistent with our research. Red represents lower values, and yellow represents higher values.

2. In the training set, the combinational model[AUC-0.900,OR-0.0282,95%CI-0.841-0.943] outperformed the clinical model[AUC-0.759,OR-0.0392,95%CI-0.683-0.825] and the imaging model[AUC-0.869,OR-0.0335,95%CI-0.804-0.918] in predictive efficacy. The decision curve confirmed that the former had a higher clinical net benefit. As above, the same results were verified in the test set[the combinational model AUC-0.856,OR-0.0494,95%CI-0.745-0.932 VS. clinical model AUC-0.766,OR-0.0640,95%CI-0.642-0.864 VS. imaging model AUC-0.782,OR-0.0597,95%CI-0.660-0.876]. It can be said that the nomogram created from the combinational model helped simplify the prediction workflow (**Figures 4**–**7**).

## Discussion

According to World Health Statistics, ESCC ranks the seventh among all cancers in the world in terms of incidence, posing severe threats to treatment and management. In China, the incidence and mortality of ESCC are about 2-3 times the global average, and approximately 400 thousand cases are newly diagnosed every year. Although the treatments and nursing procedures for ESCC have been improving continuously in China, the situation has remained grim. The postoperative 5-year survival rate is about 30-40%, and the survival quality is even worse among those with AL. Surgeries prevail as the preferred treatment for ESCC. Due to advances in minimally invasive surgeries and surgical nutritional support, postoperative rehabilitation has been accelerated, and the survival quality of ESCC patients has been improving. Nevertheless, the incidence of AL is still as high as 10%-21.2% after surgery. AL dramatically prolongs the hospital stay and raises the economic burden for patients. AL can cause severe chest infections, respiratory distress, septicopyemia, or even multiple organ failure. AL is an important risk factor for postoperative death and long-term survival quality of ESCC patients. Moreover, about 50-70% of ESCC patients receive postoperative radiotherapy, and the occurrence of AL will only delay wound healing and increase mortality. Given the above, precise preoperative evaluation of the risk of AL is indispensable for individualized diagnosis and treatment of ESCC (19, 20). It has been reported that LVI is a histopathological feature associated with the invasiveness of ESCC and defined as the presence of tumor cells in the intimal lining. LVI is usually known as lymphatic infiltration and vascular infiltration. The reported presence of cancer cell clusters in the vascular endothelial lining by postoperative pathology is crucial evidence of LVI. LVI can induce recurrence, delayed wound healing, and AL, which raises the need for enhancing preoperative neoadjuvant chemoradiotherapy (21, 22). As a conventional preoperative examination for ESCC, contrast-enhanced CT can effectively differentiate between tumors and normal esophageal tissues and therefore has important values in tumor discovery, tumor delineation, and tumor staging. CT-based radiomics has been proven related to LVI of tumors and can be used to predict the occurrence of ESCC-AL and improve the survival quality of patients.

In recent years, endoscopic minimally invasive surgery has been widely applied in surgical treatments. In particular, transthoracoscopic lobectomy, mediastinal tumor resection, and lymph node dissection have matured and enjoy the advantages of minimal invasiveness, fast postoperative recovery, and complete surgical excision. Despite all these advances, postoperative AL still occurs. In the present study, 74 out of 213 patients who received a radical resection of ESCC suffered from AL, accounting for 34.7%. This percentage is higher than what has been reported in foreign countries. The high prevalence of AL in ESCC patients may be explained by the dietary habits of Chinese people. Hot spot is a popular food in China, and many people are addicted to its unique flavor and the pleasant atmosphere of getting together when eating a hot pot. However, hot pot is usually at above 70 centigrades and is likely to scald the esophageal mucosa. Excessive consumption of condiments, such as chilies and peppers, that are usually present in large quantities in the hot pot, causes stimuli to the esophageal and gastrointestinal mucosa, inducing inflammation and bleeding. Besides, hot pot also contains large amounts of nitrite-preserved foods. Long-term exposure to these foods will induce mutational damage to esophageal tissues. Heavy alcohol consumption when eating a hot pot with friends and relatives only aggravates esophageal damage. Failure to abstain from hot pot after surgery is another reason for AL in ESCC patients (9, 23). We also confirm that the duration of surgery, surgical method, delta radscore 1/2, contrast enhancement patterns, peripheral lymph node metastasis, PTPI were another risk factors for AL. Surgeries for ESCC have a major impact on the respiratory system. The longer the duration of surgery, the higher the postoperative risk of pulmonary infections. Respiratory failure may even occur in some severe cases. resulting in decreased oxygenation capacity of the lungs and oxygen deprivation at the anastomotic site. Besides, the more severe PTPI, the more frequent the cough, which increases the anastomotic tension, mediastinal swing, and diaphragmatic movement to finally lead to AL. The present study demonstrated a much lower incidence of postoperative AL after laparothoracoscopy plus minimally invasive esophagectomy (MIE) compared with the modified Ivor-Lewis surgery or the Sweet surgery. This is because the anastomotic site in the former is usually located in the neck, where the wounds heal more rapidly and the nursing is easier. However, this procedure raises a higher requirement for surgical skills and incurs higher costs. The fast-in and fast-out pattern of enhancement indicates a higher malignancy level, while lymph node metastasis indicates higher invasiveness. Both will increase the surgical difficulty of ESCC, extend the resection scope, prolong the duration of surgery, and induce infections. Since the esophagus itself has no serosa, excessive resection of the proximal esophagus during anastomosis will reduce the blood supply to the residual esophagus, which is not conducive to anastomotic healing and further increases the incidence of AL (24–26).

Radiomics is an emerging non-invasive technique to extract high-throughput radiomics features from multimodality medical images. These features are used for in-depth data mining and objective assessment of tumor heterogeneity, which further informs molecular staging and differential diagnosis of tumors, treatment option selection, and prognostic evaluation. In the present study, 1065x3 radiomics features were extracted from the tumor ROI on the contrast-enhanced CT image. After the removal of redundant features, 879x3 radiomics features were finally preserved, including the morphological feature Mean…210, the Gaussian Laplace transform feature RunVariance…735, GrayLevelNonUniformity…739, and wavelet feature MCC…239. Mean…210 is a radiomics shape feature that can describe the differences between the tumor volume and a perfect cube. This feature is independent of the segmentation method but related to the tumor volume. The larger the tumor volume, the smaller the value. We found that ESCC with a lower Mean…210 was associated with a higher risk of AL, thus highlighting the importance of the maximal diameter of ESCC for AL prediction. Laplace transform is an edge enhancement filter that highlights regions with grayscale variation and textural non-uniformity. More valuable radiomics features can be extracted from the raw images using Laplace transform to more correctly characterize the heterogeneity and LVI of ESCC and enhance the predictive performance for AL. Delta-Radscore1/2 were found to be potent predictors for AL for the first time in this study. These High-quality datas on enhanced CT perfusion imaging reveal more details about the nature of ESCC. Delta-Radscore1/2 exploits the differentiation of radiomics features before and after the contrast-enhanced CT, offering valuable clues for measuring blood supply and intratumoral composition of ESCC. Delta-Radscore1/2 reveal the malignant potential of ESCC and help make clinical decisions. At present, Delta-radiomics plays an important role in the postoperative management of head and neck tumors and surpasses static radiomics in many aspects. In addition, the above-mentioned radiomics features are often related to classical imaging features such as global hypodensity, heterogeneity, different CT values, tissue necrosis, blurred boundaries, tumor enhancement patterns, and surrounding tissue infiltration, which may help identify subtle imaging features that cannot be distinguished by the human naked eye and enhance the differentiation of tumor malignancy potential. In this study, we combined Delta Radscore with clinical data when building a predictive model for AL, which exhibits a higher predictive performance than most of the previous ones. It was not only because the contrast-enhanced CT offers more abundant low-level and underlying radiological features of ESCC, but more importantly, the Delta Radscore quantifies the differentiation of radiomics textures of ESCC and reflects the intrinsic properties of ESCC (27–29).

## Limitations

Although the study included a reasonable number of ESCC patients (n = 213), it is conducted at a single center. The results may not be generalizable to other populations or clinical settings. A multi-center study with a larger cohort would enhance the generalizability of the findings. Errors are inevitable in the manual delineation of ROI. The images of plain MR scans and multi-phase contrast-enhanced MR scans were not included, and we might have missed some significant variables. We did not evaluate the influence of AL on the prognosis of ESCC patients using radiomics, which will be our research focus in the future (30, 31).

## Conclusions

To conclude, delta-radscore 1/2, PTPI, hot pot are an important risk factors for ESCC-AL. A novel nomogram created based on the above factors is effective in preoperative assessment of AL, helps optimize treatment decisions for ESCC, and promotes perioperative nursing. This nomogram provides data support for the individualized treatment of ESCC-AL.

## Data Availability

The original contributions presented in the study are included in the article/supplementary material. Further inquiries can be directed to the corresponding authors.

## References

[1] Shu WY Shi YY Huang JT Meng LM Zhang HJ Cui RL. Microvascular structural changes in esophageal squamous cell carcinoma pathology according to intrapapillary capillary loop types under magnifying endoscopy. World J Gastrointest Oncol. (2024) 16:3471–80. doi: 10.4251/wjgo.v16.i8.3471 PMC11334018 39171175

[2] Pomenti SF Flashner SP Del Portillo A Nakagawa H Gabre J Rustgi AK. Clinical and biological perspectives on non-canonical esophageal squamous cell carcinoma in rare subtypes. Am J Gastroenterol. (2024). doi: 10.14309/ajg.0000000000003041 39166765

[3] Lu Q Yang Q Zhao J Li G Zhang J Jia C. The identification of heterogeneous reactive oxygen subtypes in esophageal squamous cell carcinoma to aid patient prognosis and immunotherapy. Heliyon. (2024) 10:e35235. doi: 10.1016/j.heliyon.2024.e35235 39165982 PMC11334838

[4] Guo Y Wang T Liu Y Gu D Li H Liu Y. Comparison of immunochemotherapy followed by surgery or chemoradiotherapy in locally advanced esophageal squamous cell cancer. Int Immunopharmacol. (2024) 141:112939. doi: 10.1016/j.intimp.2024.112939 39151385

[5] Jeon S Park SY. ASO author reflections: the timeless debate between ivor-lewis or mcKeown esophagectomy. Ann Surg Oncol. (2024). doi: 10.1245/s10434-024-16007-z 39107602

[6] Zhou X Yue H Zheng Z Zhang W Wang J Peng L. Predicting pathological response in esophageal squamous cell carcinoma with longitudinal CT radiomics and disentangled representation learning: a multicenter retrospective cohort study. Int J Surg. (2024). doi: 10.1097/JS9.0000000000001985 PMC11745662 39051670

[7] Liu Y Ma Z Bao Y Wang X Men Y Sun X. Integrating MR radiomics and dynamic hematological factors predicts pathological response to neoadjuvant chemoradiotherapy in esophageal cancer. Heliyon. (2024) 10:e33702. doi: 10.1016/j.heliyon.2024.e33702 39050414 PMC11268188

[8] Xie SH Zhang WF Wu Y Tang ZL Yang LT Xue YJ. Application of predictive model based on CT radiomics and machine learning in diagnosis for occult locally advanced esophageal squamous cell carcinoma before treatment: A two-center study. Transl Oncol. (2024) 47:102050. doi: 10.1016/j.tranon.2024.102050 38981245 PMC11292555

[9] Liu J Song L Zhou J Yu M Hu Y Zhang J. Prediction of prognosis of tongue squamous cell carcinoma based on clinical MR imaging data modeling. Technol Cancer Res Treat. (2023) 22:15330338231207006. doi: 10.1177/15330338231207006 37872687 PMC10594972

[10] Shen D Chen R Wu Q Ji Y van der Wilk BJ Chen EY. Safety and short-term outcomes of esophagectomy after neoadjuvant immunotherapy combined with chemotherapy or chemoradiotherapy for locally advanced esophageal squamous cell cancer: analysis of two phase-II clinical trials. J Gastrointest Oncol. (2024) 15:841–50. doi: 10.21037/jgo-24-295 PMC11231833 38989436

[11] Shi K Qian R Zhang X Jin Z Lin T Lang B. Video-assisted mediastinoscopic and laparoscopic transhiatal esophagectomy for esophageal cancer. Surg Endosc. (2022) 36:4207–14. doi: 10.1007/s00464-021-08754-x 34642798

[12] Sarkaria IS Rizk NP Finley DJ Bains MS Adusumilli PS Huang J. Combined thoracoscopic and laparoscopic robotic-assisted minimally invasive esophagectomy using a four-arm platform: experience, technique and cautions during early procedure development. Eur J Cardiothorac Surg. (2013) 43:e107–15. doi: 10.1093/ejcts/ezt013 23371971

[13] Zeng F He B Wang Y Xue Y Cong W. Combined thoracoscopic and laparoscopic minimally invasive esophagectomy. J Thorac Dis. (2014) 6:152–5. doi: 10.3978/j.issn.2072-1439.2014.02.04 PMC3944189 24605230

[14] Kamarajah SK Markar SR. Navigating complexities and considerations for suspected anastomotic leakage in the upper gastrointestinal tract: A state of the art review. Best Pract Res Clin Gastroenterol. (2024) 70:101916. doi: 10.1016/j.bpg.2024.101916 39053974

[15] Li Y Su X Shang Y Liu H Wang W Zhang A. Comparative evaluation of imaging methods for prognosis assessment in esophageal squamous cell carcinoma: focus on diffusion-weighted magnetic resonance imaging, computed tomography and esophagography. Front Oncol. (2024) 14:1397266. doi: 10.3389/fonc.2024.1397266 39026975 PMC11256006

[16] Fan L Yang Z Chang M Chen Z Wen Q. CT-based delta-radiomics nomogram to predict pathological complete response after neoadjuvant chemoradiotherapy in esophageal squamous cell carcinoma patients. J Transl Med. (2024) 22:579. doi: 10.1186/s12967-024-05392-4 38890720 PMC11186275

[17] Wang JL Tang LS Zhong X Wang Y Feng YJ Zhang Y. A machine learning radiomics based on enhanced computed tomography to predict neoadjuvant immunotherapy for resectable esophageal squamous cell carcinoma. Front Immunol. (2024) 15:1405146. doi: 10.3389/fimmu.2024.1405146 38947338 PMC11211602

[18] Huang J Li T Tang L Hu Y Hu Y Gu Y. Development and validation of an 18F-FDG PET/CT-based radiomics nomogram for predicting the prognosis of patients with esophageal squamous cell carcinoma. Acad Radiol. (2024), S1076–6332(24)00335-0. doi: 10.1016/j.acra.2024.05.029 38845294

[19] Chevallay M Jung M Chon SH Takeda FR Akiyama J Mönig S. Esophageal cancer surgery: review of complications and their management. Ann N Y Acad Sci. (2020) 1482:146–62. doi: 10.1111/nyas.14492 32935342

[20] Chen XF Lin JP Zhou H Kang BZ Nayak R Gao L. The relationship between the collagen score at the anastomotic site of esophageal squamous cell carcinoma and anastomotic leakage. J Thorac Dis. (2024) 16:4515–24. doi: 10.21037/jtd-24-427 PMC11320265 39144302

[21] Li Y Yu M Wang G Yang L Ma C Wang M. Contrast-enhanced CT-based radiomics analysis in predicting lymphovascular invasion in esophageal squamous cell carcinoma. Front Oncol. (2021) 11:644165. doi: 10.3389/fonc.2021.644165 34055613 PMC8162215

[22] Miyata H Sugimura K Kanemura T Takeoka T Sugase T Yasui M. Prognostic impact of nodal status and lymphovascular invasion in patients undergoing neoadjuvant chemotherapy for esophageal squamous cell carcinoma. Dis Esophagus. (2024), doae038. doi: 10.1093/dote/doae038 38693752

[23] Huang X Han D Cheng J Chen X Zhou Y Liao H. Characteristics and health risk assessment of volatile organic compounds (VOCs) in restaurants in Shanghai. Environ Sci pollut Res Int. (2020) 27:490–9. doi: 10.1007/s11356-019-06881-6 31797266

[24] Wang YJ He XD He YQ Bao T Xie XF Li KK. Comparison of two different methods for lymphadenectomy along the left recurrent laryngeal nerve by minimally invasive esophagectomy in patients with esophageal squamous cell carcinoma: a prospective randomized trial. Int J Surg. (2024) 110:159–66. doi: 10.1097/JS9.0000000000000788 PMC10793764 37737902

[25] Raftery NB Murphy CF Donohoe CL O'Connell B King S Ravi N. The complexity of defining postoperative pneumonia after esophageal cancer surgery: A spectrum of lung injury rather than a simple infective complication? Ann Surg. (2022) 276:e400–6. doi: 10.1097/SLA.0000000000004546 33201133

[26] Lin F Zhu LX Ye ZM Peng F Chen MC Li XM. Computed tomography-based intratumor heterogeneity predicts response to immunotherapy plus chemotherapy in esophageal squamous cell carcinoma. Acad Radiol. (2024), S1076–6332(24)00418-5. doi: 10.1016/j.acra.2024.06.032 38981774

[27] Huang YC Huang SM Yeh JH Chang TC Tsan DL Lin CY. Utility of CT radiomics and delta radiomics for survival evaluation in locally advanced nasopharyngeal carcinoma with concurrent chemoradiotherapy. Diagnostics (Basel). (2024) 14:941. doi: 10.3390/diagnostics14090941 38732355 PMC11083477

[28] Zhang AD Shi QL Zhang HT Duan WH Li Y Ruan L. Pairwise machine learning-based automatic diagnostic platform utilizing CT images and clinical information for predicting radiotherapy locoregional recurrence in elderly esophageal cancer patients. Abdom Radiol (NY). (2024). doi: 10.1007/s00261-024-04377-7 PMC11519085 38831075

[29] Kawahara D Nishioka R Murakami Y Emoto Y Iwashita K Sasaki R. A nomogram based on pretreatment radiomics and dosiomics features for predicting overall survival associated with esophageal squamous cell cancer. Eur J Surg Oncol. (2024) 50:108450. doi: 10.1016/j.ejso.2024.108450 38843660

[30] Hu HH Xu X Li XY Zeng Y Li Y Song XY. The value of intervention with radiotherapy after first-line chemo-immunotherapy in locally advanced or metastatic esophageal squamous cell carcinoma: A multi-center retrospective study. Clin Transl Radiat Oncol. (2024) 48:100818. doi: 10.1016/j.ctro.2024.100818 39091465 PMC11292253

[31] Shi YJ Yan S Yang X Guan Z Li XT Wang LL. Early contrast-enhanced MR for diagnosing complete tumor response of locally advanced esophageal squamous cell carcinoma after neoadjuvant therapy: A retrospective comparative study. Ann Surg Oncol. (2024) 31:4271–80. doi: 10.1245/s10434-024-15123-0 38453768

